# Supplementary material for: Human Anti-Als3p Antibodies Are Surrogate Markers of NDV-3A Vaccine Efficacy Against Recurrent Vulvovaginal Candidiasis
Source: Front Immunol. 2018 Jun 15;9:1349. doi: 10.3389/fimmu.2018.01349 (PMC6013566; doi:10.3389/fimmu.2018.01349)
Supplement: Supplementary file 1 [file presentation_1.PPTX]

## Slide 1
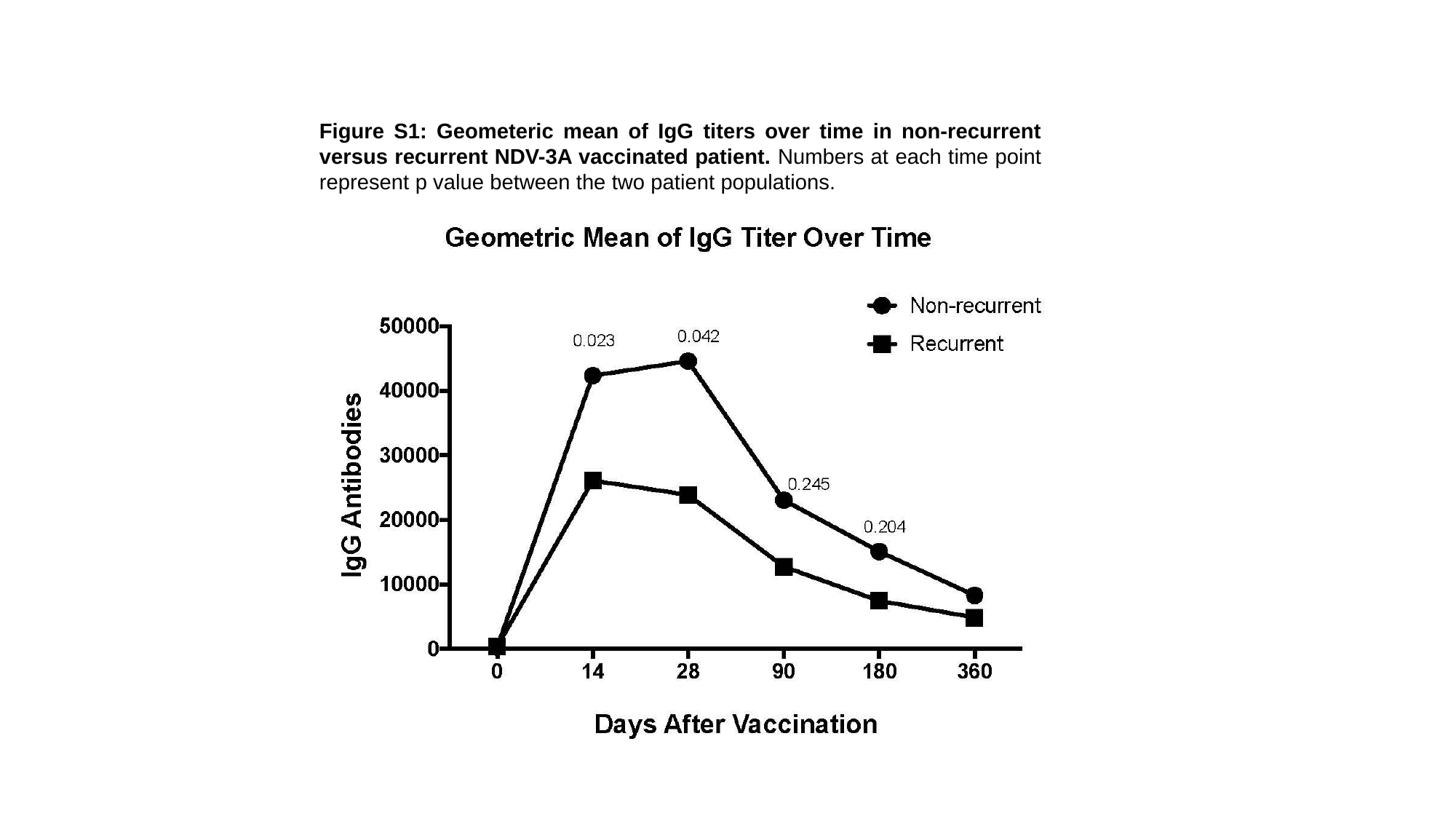

Figure S1: Geometeric mean of IgG titers over time in non-recurrent versus recurrent NDV-3A vaccinated patient. Numbers at each time point represent p value between the two patient populations.

## Slide 2
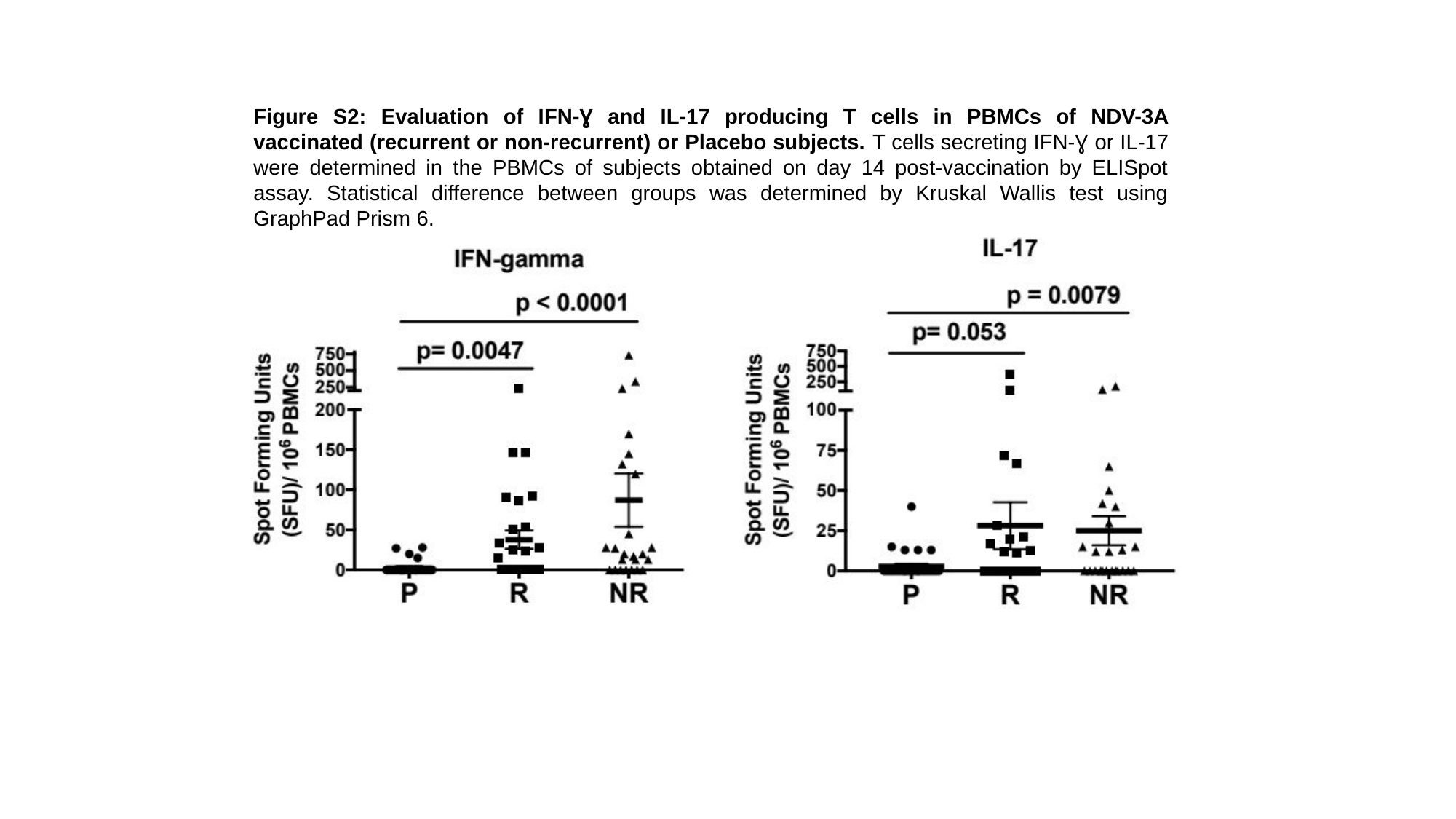

Figure S2: Evaluation of IFN-Ɣ and IL-17 producing T cells in PBMCs of NDV-3A vaccinated (recurrent or non-recurrent) or Placebo subjects. T cells secreting IFN-Ɣ or IL-17 were determined in the PBMCs of subjects obtained on day 14 post-vaccination by ELISpot assay. Statistical difference between groups was determined by Kruskal Wallis test using GraphPad Prism 6.

## Slide 3
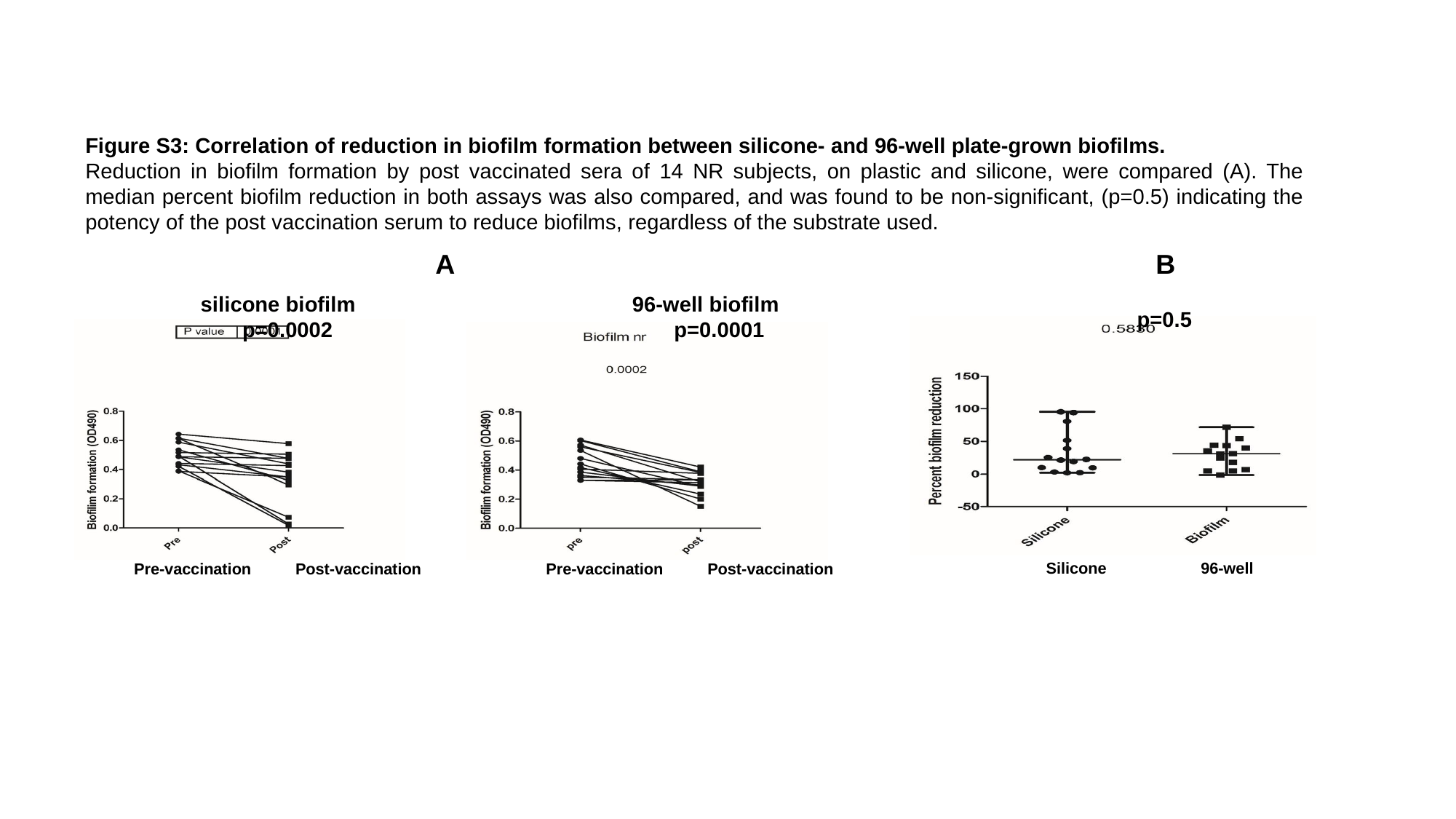

Figure S3: Correlation of reduction in biofilm formation between silicone- and 96-well plate-grown biofilms.
Reduction in biofilm formation by post vaccinated sera of 14 NR subjects, on plastic and silicone, were compared (A). The median percent biofilm reduction in both assays was also compared, and was found to be non-significant, (p=0.5) indicating the potency of the post vaccination serum to reduce biofilms, regardless of the substrate used.
A
B
silicone biofilm
p=0.0002
Pre-vaccination
Post-vaccination
96-well biofilm
p=0.0001
Pre-vaccination
Post-vaccination
p=0.5
Silicone
96-well

## Slide 4
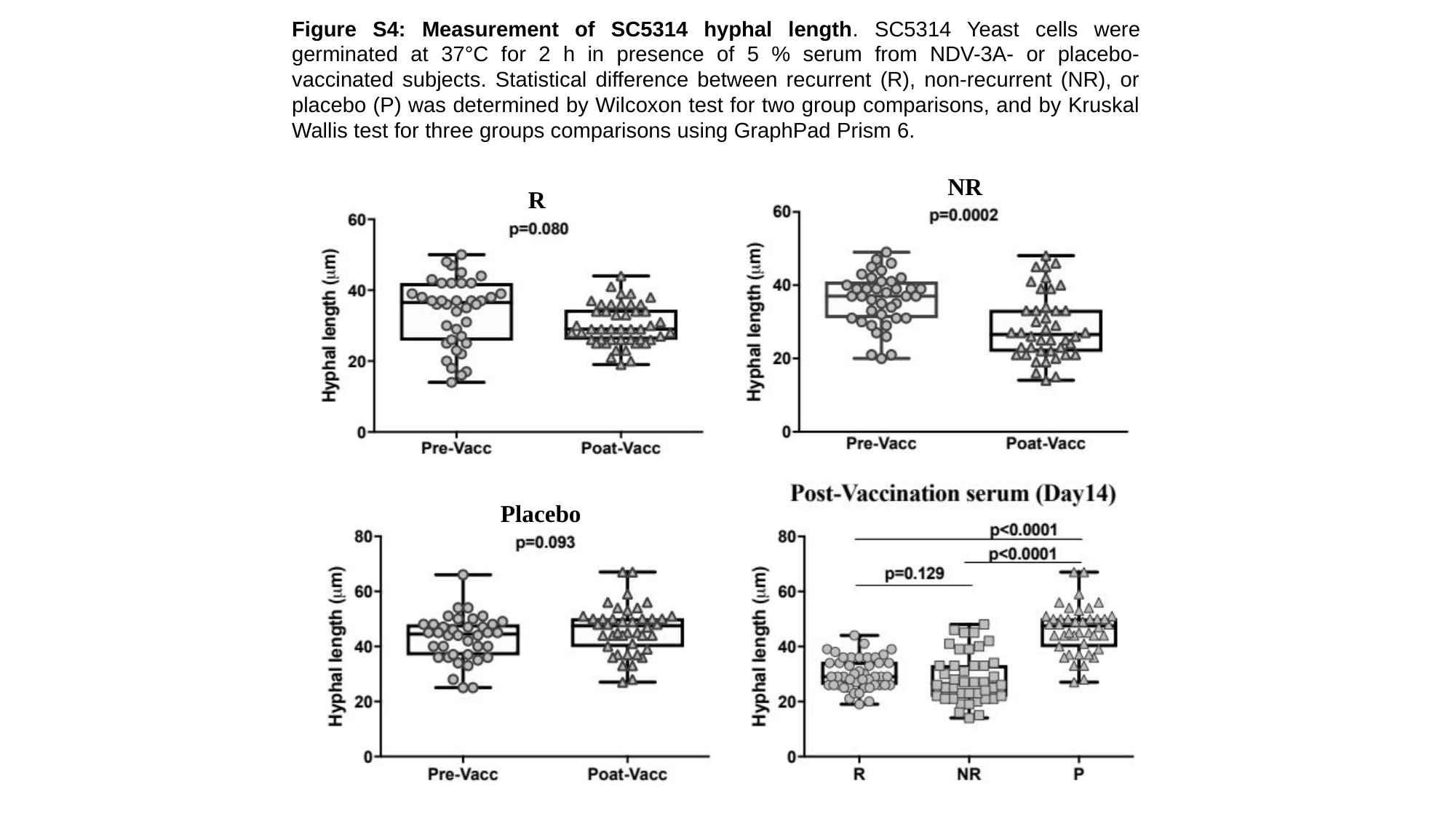

Figure S4: Measurement of SC5314 hyphal length. SC5314 Yeast cells were germinated at 37°C for 2 h in presence of 5 % serum from NDV-3A- or placebo-vaccinated subjects. Statistical difference between recurrent (R), non-recurrent (NR), or placebo (P) was determined by Wilcoxon test for two group comparisons, and by Kruskal Wallis test for three groups comparisons using GraphPad Prism 6.
NR
R
Placebo

## Slide 5
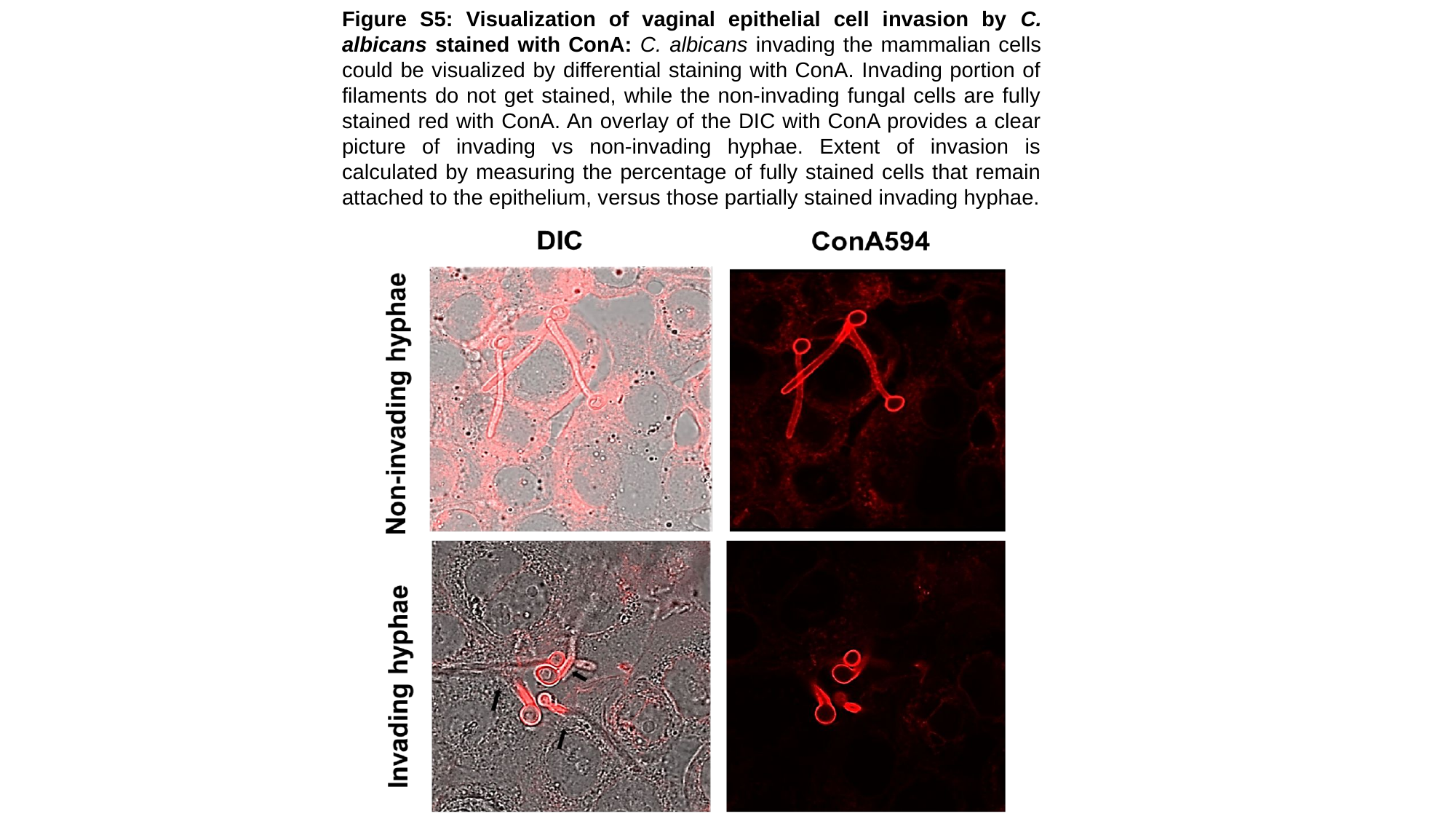

Figure S5: Visualization of vaginal epithelial cell invasion by C. albicans stained with ConA: C. albicans invading the mammalian cells could be visualized by differential staining with ConA. Invading portion of filaments do not get stained, while the non-invading fungal cells are fully stained red with ConA. An overlay of the DIC with ConA provides a clear picture of invading vs non-invading hyphae. Extent of invasion is calculated by measuring the percentage of fully stained cells that remain attached to the epithelium, versus those partially stained invading hyphae.

## Slide 6
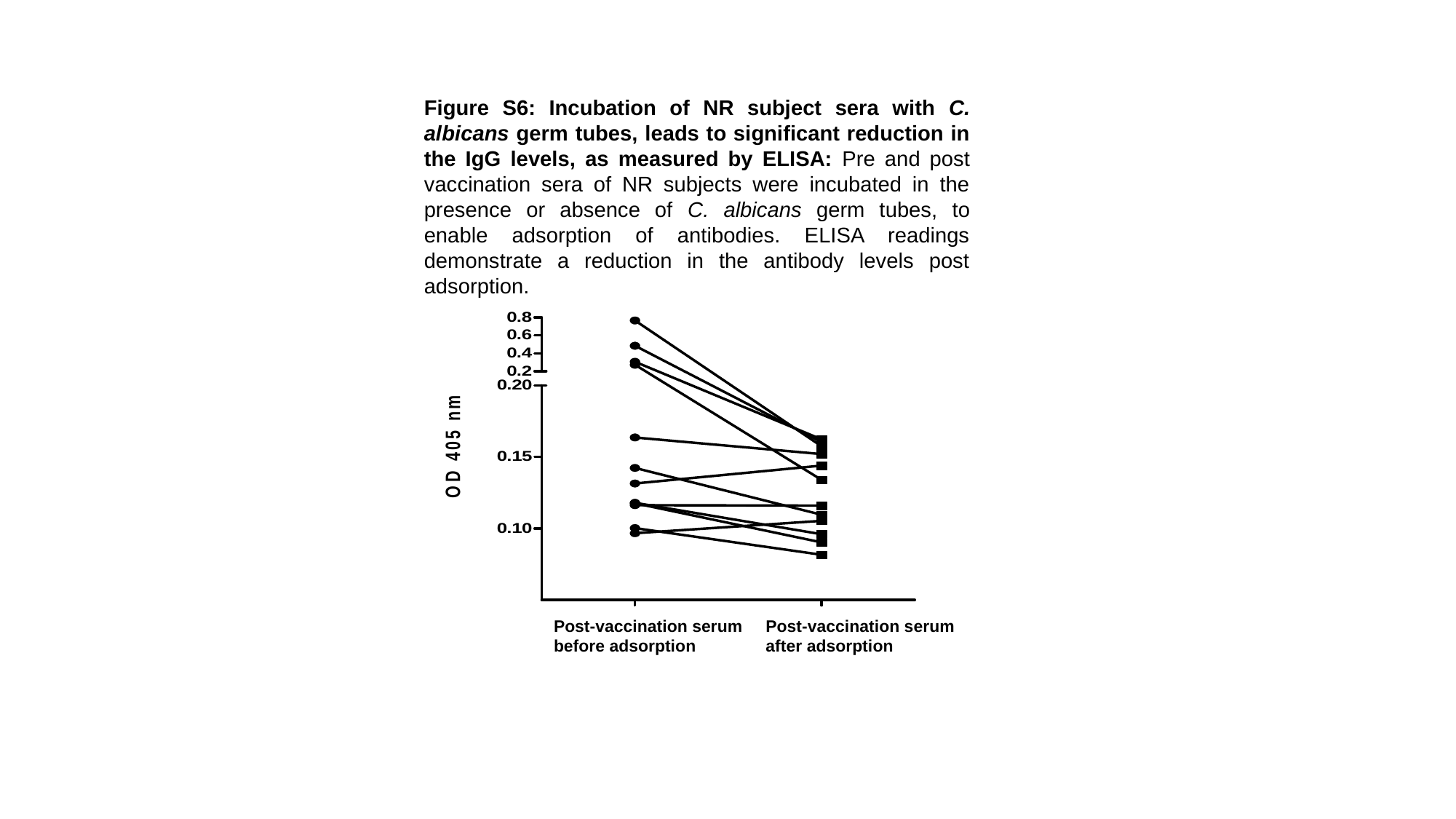

Figure S6: Incubation of NR subject sera with C. albicans germ tubes, leads to significant reduction in the IgG levels, as measured by ELISA: Pre and post vaccination sera of NR subjects were incubated in the presence or absence of C. albicans germ tubes, to enable adsorption of antibodies. ELISA readings demonstrate a reduction in the antibody levels post adsorption.
Post-vaccination serum
after adsorption
Post-vaccination serum
before adsorption

## Slide 7
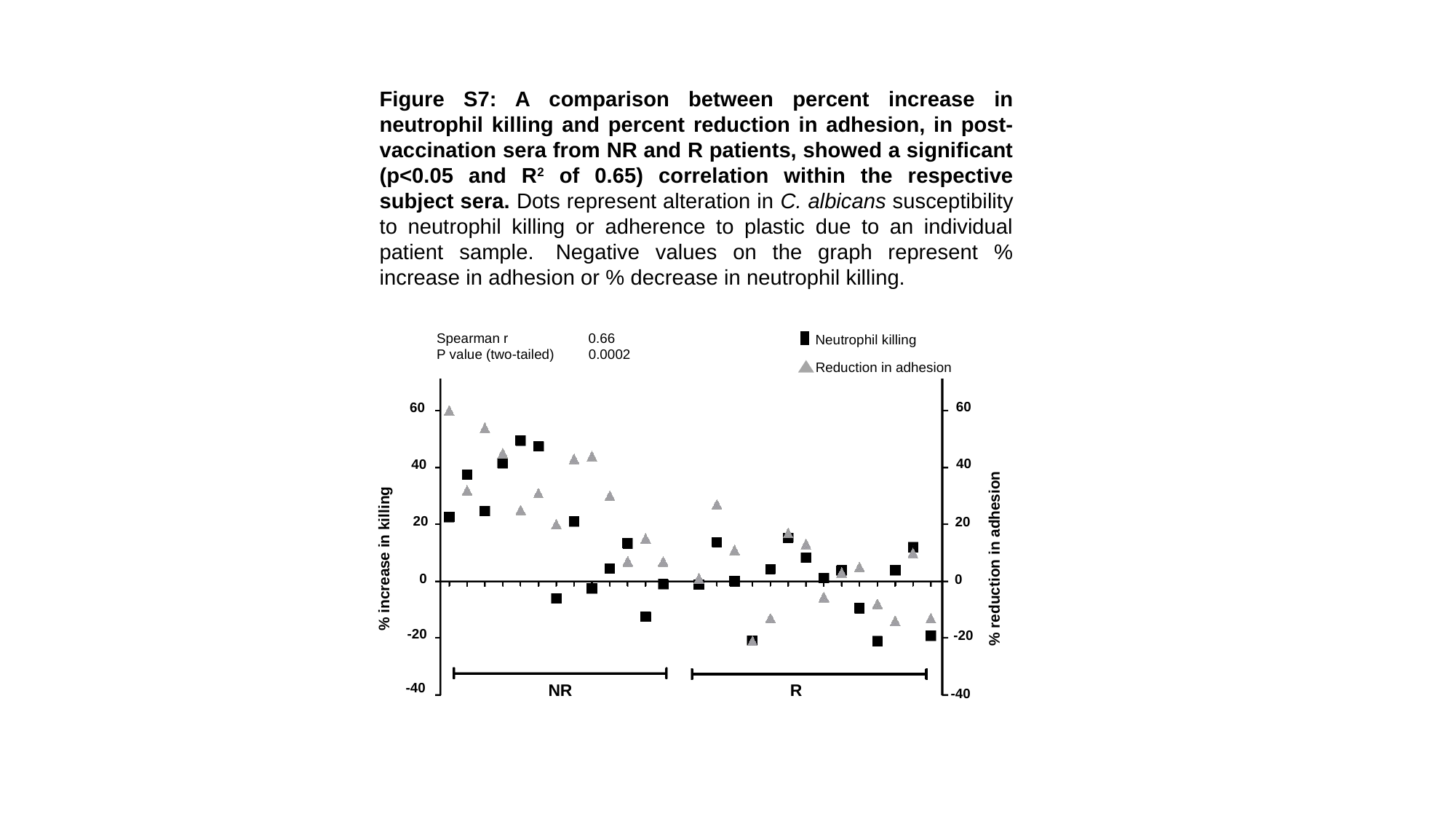

Figure S7: A comparison between percent increase in neutrophil killing and percent reduction in adhesion, in post-vaccination sera from NR and R patients, showed a significant (p<0.05 and R2 of 0.65) correlation within the respective subject sera. Dots represent alteration in C. albicans susceptibility to neutrophil killing or adherence to plastic due to an individual patient sample.  Negative values on the graph represent % increase in adhesion or % decrease in neutrophil killing.
Spearman r 0.66
P value (two-tailed) 0.0002
Neutrophil killing
Reduction in adhesion
 60
 60
 40
 40
% increase in killing
% reduction in adhesion
 20
 20
 0
 0
-20
-20
-40
NR
R
-40
